# *catena*-Poly[[(1,10-phenanthroline)cadmium(II)]-μ-2-(1,3-benzimidazol-2-ylsulfan­yl)acetato-κ^3^ *N* ^1^,*O*:*N* ^3^]

**DOI:** 10.1107/S1600536808041044

**Published:** 2008-12-10

**Authors:** Lin Cheng, Yan-Yan Sun, Ya-Wen Zhang, Jian-Quan Wang

**Affiliations:** a School of Chemistry and Chemical Engineering, Southeast University, Nanjing 211189, People’s Republic of China

## Abstract

In title compound, [Cd(C_9_H_6_N_2_O_2_S)(C_12_H_8_N_2_)]_*n*_, the Cd^II^ atom is in a distorted tetra­gonal-pyramidal environment, coordinated by one chelating 1,10-phenanthroline ligand, one chelating 2-(1,3-benzimidazol-2-ylsulfan­yl)acetate (bia) ligand bound through one N atom and one O atom of the carboxyl group, and one N atom from a second bia ligand. Each bia ligand acts as bridge between Cd^II^ ions, forming one-dimensional coordination polymers along [010], with a shortest Cd⋯Cd distance of 4.27 (2) Å.

## Related literature

For related structures, see: Matthews *et al.* (1998 ▶); Zhang *et al.* (2008 ▶).
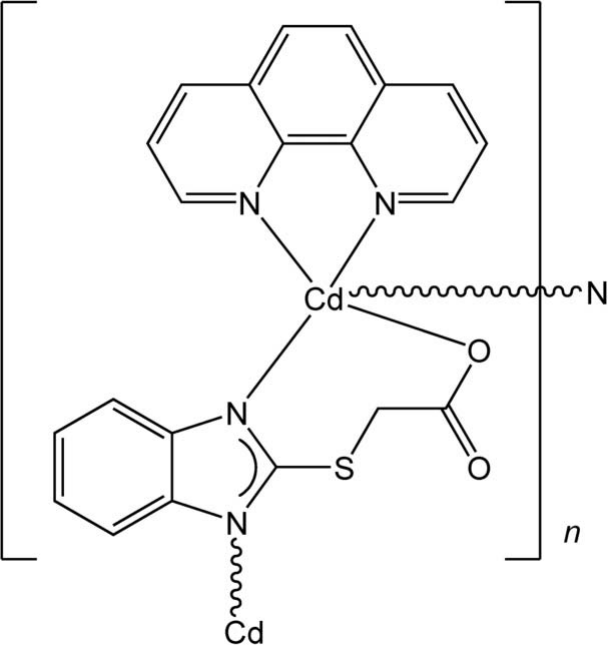


## Experimental

### 

#### Crystal data

[Cd(C_9_H_6_N_2_O_2_S)(C_12_H_8_N_2_)]
*M* *_r_* = 498.84
Monoclinic, 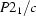

*a* = 9.2195 (10) Å
*b* = 8.2577 (9) Å
*c* = 25.620 (3) Å
β = 103.215 (4)°
*V* = 1898.8 (4) Å^3^
*Z* = 4
Mo *K*α radiation
μ = 1.29 mm^−1^
*T* = 293 (2) K
0.20 × 0.18 × 0.15 mm

#### Data collection

Bruker APEX CCD diffractometer
Absorption correction: multi-scan (*SADABS*; Sheldrick, 2000 ▶) *T*_min_ = 0.783, *T*_max_ = 0.831
9820 measured reflections
3724 independent reflections
3413 reflections with *I* > 2σ(*I*)
*R*_int_ = 0.025

#### Refinement

*R*[*F* ^2^ > 2σ(*F* ^2^)] = 0.041
*wR*(*F* ^2^) = 0.091
*S* = 1.04
3724 reflections
262 parameters
H-atom parameters constrained
Δρ_max_ = 0.62 e Å^−3^
Δρ_min_ = −0.46 e Å^−3^

### 

Data collection: *SMART* (Bruker, 2000 ▶); cell refinement: *SAINT* (Bruker, 2000 ▶); data reduction: *SAINT*; program(s) used to solve structure: *SHELXTL* (Sheldrick, 2008 ▶); program(s) used to refine structure: *SHELXTL*; molecular graphics: *SHELXTL*; software used to prepare material for publication: *SHELXTL*.

## Supplementary Material

Crystal structure: contains datablocks I, global. DOI: 10.1107/S1600536808041044/bi2328sup1.cif

Structure factors: contains datablocks I. DOI: 10.1107/S1600536808041044/bi2328Isup2.hkl

Additional supplementary materials:  crystallographic information; 3D view; checkCIF report

## Figures and Tables

**Fig. 1.**
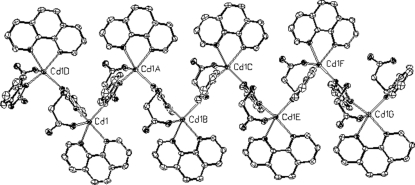
The one-dimensional chain of the title compound. Displacement ellipsoids are shown at 50% probability and H atoms are omitted for clarity.

## supplementary crystallographic information

### Comment

Recently, there has been significant interest in the rational design and synthesis of metal-organic coordination architectures by using flexible bridging units because the flexibility and conformational freedom of such ligands offers the possibility for construction of unprecedented frameworks (Zhang *et al.* 2008). Benzimidazole and thioether carboxylates have been widely used to construct many novel and interesting metal-organic frameworks. However, such metal-organic frameworks formed by bifunctional ligands including benzimidazole and thioether carboxylate have been rarely reported (Matthews *et al.* 1998). Herein, we report a new bifunctional flexible ligand 2-(1*H*-benzo[*d*]imidazol-2-ylthio)acetic acid (H_2_bia), and present the synthesis and structural characterization of a one-dimensional coordination polymer {Cd(bia)(phen)}*n* (phen = 1,10-phenanthroline).

The asymmetric unit of the title compound, contains a Cd^II^ cation, a bia and a chelating phen. Each Cd^II^ displays a distorted tetragonal pyramidal geometry, being surrounded by one phen ligand, one chelating bia and a N atom of another bia ligand. Each bia ligand acts as a bridge between two Cd^II^ ions, forming one-dimensional coordination polymers along the *b* axis (Fig. 1). The shortest Cd···Cd distance in the chain is 4.27 (2) Å.

### Experimental

A mixture of H_2_bia (0.0208 g, 0.1 mmol), phen (0.0180 g, 0.1 mmol), Cd(NO_3_)_2_.6H_2_O (0.0345 g, 0.1 mmol) and H_2_O (8 ml) was heated in a 15 ml Teflon-lined autoclave at 433 K for 3 days, followed by slow cooling (5 K h^-1^) to room temperature. The resulting mixture was washed with water, and colourless block crystals were collected and dried in air. Yield 86% (42.9 mg), based on Mn^II^.

### Refinement

H atoms were positioned geometrically and refined using a riding model with C—H = 0.93 or 0.97 Å and with *U*_iso_(H) = 1.2*U*_eq_(C).

### Crystal data

**Table:**

[Cd(C_9_H_6_N_2_O_2_S)(C_12_H_8_N_2_)]	*F*(000) = 992
*M**_r_* = 498.84	*D*_x_ = 1.745 Mg m^−^^3^
Monoclinic, *P*2_1_/*c*	Mo *K*α radiation, λ = 0.71073 Å
Hall symbol: -P 2ybc	Cell parameters from 781 reflections
*a* = 9.2195 (10) Å	θ = 2.4–28.0°
*b* = 8.2577 (9) Å	µ = 1.29 mm^−^^1^
*c* = 25.620 (3) Å	*T* = 293 K
β = 103.215 (4)°	Block, colorless
*V* = 1898.8 (4) Å^3^	0.20 × 0.18 × 0.15 mm
*Z* = 4	

### Data collection

**Table:**

Bruker APEX CCD diffractometer	3724 independent reflections
Radiation source: fine-focus sealed tube	3413 reflections with *I* > 2σ(*I*)
graphite	*R*_int_ = 0.025
φ and ω scans	θ_max_ = 26.0°, θ_min_ = 2.3°
Absorption correction: multi-scan (*SADABS*; Sheldrick, 2000)	*h* = −11→11
*T*_min_ = 0.783, *T*_max_ = 0.831	*k* = −9→10
9820 measured reflections	*l* = −31→26

### Refinement

**Table:**

Refinement on *F*^2^	Primary atom site location: structure-invariant direct methods
Least-squares matrix: full	Secondary atom site location: difference Fourier map
*R*[*F*^2^ > 2σ(*F*^2^)] = 0.041	Hydrogen site location: inferred from neighbouring sites
*wR*(*F*^2^) = 0.091	H-atom parameters constrained
*S* = 1.04	*w* = 1/[σ^2^(*F*_o_^2^) + (0.0401*P*)^2^ + 3.0611*P*] where *P* = (*F*_o_^2^ + 2*F*_c_^2^)/3
3724 reflections	(Δ/σ)_max_ < 0.001
262 parameters	Δρ_max_ = 0.62 e Å^−^^3^
0 restraints	Δρ_min_ = −0.46 e Å^−^^3^

### Special details

**Table:**

Geometry. All e.s.d.'s (except the e.s.d. in the dihedral angle between two l.s. planes) are estimated using the full covariance matrix. The cell e.s.d.'s are taken into account individually in the estimation of e.s.d.'s in distances, angles and torsion angles; correlations between e.s.d.'s in cell parameters are only used when they are defined by crystal symmetry. An approximate (isotropic) treatment of cell e.s.d.'s is used for estimating e.s.d.'s involving l.s. planes.
Refinement. Refinement of *F*^2^ against ALL reflections. The weighted *R*-factor *wR* and goodness of fit *S* are based on *F*^2^, conventional *R*-factors *R* are based on *F*, with *F* set to zero for negative *F*^2^. The threshold expression of *F*^2^ > σ(*F*^2^) is used only for calculating *R*-factors(gt) *etc*. and is not relevant to the choice of reflections for refinement. *R*-factors based on *F*^2^ are statistically about twice as large as those based on *F*, and *R*- factors based on ALL data will be even larger.

### Fractional atomic coordinates and isotropic or equivalent isotropic displacement parameters (Å^2^)

**Table:**

	*x*	*y*	*z*	*U*_iso_*/*U*_eq_	
Cd1	−0.02266 (3)	−0.35415 (3)	−0.160159 (10)	0.03702 (11)	
S1	−0.16960 (11)	−0.61304 (14)	−0.27062 (4)	0.0438 (2)	
O1	−0.2554 (4)	−0.4866 (4)	−0.42417 (12)	0.0656 (9)	
O2	−0.1646 (3)	−0.7175 (4)	−0.38778 (11)	0.0536 (8)	
N1	0.0906 (3)	−0.5378 (4)	−0.20121 (12)	0.0375 (7)	
N2	0.1161 (3)	−0.6933 (4)	−0.27140 (12)	0.0382 (7)	
N3	−0.2011 (4)	−0.2403 (4)	−0.11518 (12)	0.0420 (8)	
N4	−0.0690 (3)	−0.5365 (4)	−0.09663 (12)	0.0372 (7)	
C1	0.3638 (5)	−0.5327 (6)	−0.15550 (18)	0.0543 (11)	
H1A	0.3548	−0.4674	−0.1268	0.065*	
C2	0.4989 (5)	−0.5924 (7)	−0.1596 (2)	0.0638 (13)	
H2A	0.5829	−0.5674	−0.1331	0.077*	
C3	0.5142 (5)	−0.6896 (6)	−0.2025 (2)	0.0578 (12)	
H3A	0.6080	−0.7285	−0.2038	0.069*	
C4	0.3944 (4)	−0.7286 (6)	−0.24257 (17)	0.0481 (10)	
H4A	0.4049	−0.7929	−0.2713	0.058*	
C5	0.2560 (4)	−0.6688 (5)	−0.23888 (15)	0.0369 (8)	
C6	0.2405 (4)	−0.5729 (5)	−0.19558 (15)	0.0382 (8)	
C7	0.0246 (4)	−0.6129 (4)	−0.24705 (14)	0.0350 (8)	
C8	−0.1951 (5)	−0.4915 (5)	−0.33087 (16)	0.0503 (10)	
H8A	−0.2856	−0.4290	−0.3335	0.060*	
H8B	−0.1133	−0.4147	−0.3255	0.060*	
C9	−0.2050 (4)	−0.5725 (5)	−0.38529 (15)	0.0401 (9)	
C10	−0.2683 (5)	−0.0977 (6)	−0.12479 (18)	0.0533 (11)	
H10A	−0.2493	−0.0352	−0.1527	0.064*	
C11	−0.3653 (5)	−0.0372 (6)	−0.09554 (19)	0.0600 (12)	
H11A	−0.4117	0.0624	−0.1042	0.072*	
C12	−0.3913 (5)	−0.1260 (6)	−0.05392 (18)	0.0577 (12)	
H12A	−0.4549	−0.0865	−0.0335	0.069*	
C13	−0.3225 (4)	−0.2768 (6)	−0.04181 (16)	0.0443 (9)	
C14	−0.3439 (5)	−0.3777 (6)	0.00100 (18)	0.0551 (12)	
H14A	−0.4031	−0.3409	0.0234	0.066*	
C15	−0.2810 (5)	−0.5237 (6)	0.00960 (17)	0.0519 (11)	
H15A	−0.2986	−0.5873	0.0375	0.062*	
C16	−0.1871 (4)	−0.5838 (5)	−0.02325 (15)	0.0400 (9)	
C17	−0.1199 (5)	−0.7361 (6)	−0.01553 (16)	0.0480 (10)	
H17A	−0.1378	−0.8042	0.0112	0.058*	
C18	−0.0281 (5)	−0.7844 (5)	−0.04729 (17)	0.0497 (10)	
H18A	0.0185	−0.8849	−0.0423	0.060*	
C19	−0.0053 (5)	−0.6804 (5)	−0.08740 (16)	0.0450 (10)	
H19A	0.0579	−0.7139	−0.1088	0.054*	
C20	−0.1588 (4)	−0.4874 (5)	−0.06459 (14)	0.0351 (8)	
C21	−0.2282 (4)	−0.3303 (5)	−0.07459 (14)	0.0372 (8)	

### Atomic displacement parameters (Å^2^)

**Table:**

	*U*^11^	*U*^22^	*U*^33^	*U*^12^	*U*^13^	*U*^23^
Cd1	0.04657 (18)	0.03648 (18)	0.02956 (16)	0.00219 (12)	0.01194 (11)	0.00272 (11)
S1	0.0372 (5)	0.0561 (6)	0.0393 (5)	−0.0041 (5)	0.0109 (4)	−0.0040 (5)
O1	0.093 (3)	0.056 (2)	0.0449 (18)	0.0063 (18)	0.0098 (17)	0.0128 (15)
O2	0.0666 (19)	0.0493 (18)	0.0396 (16)	0.0129 (15)	0.0011 (14)	−0.0080 (13)
N1	0.0437 (18)	0.0410 (18)	0.0279 (15)	−0.0012 (14)	0.0085 (13)	−0.0034 (13)
N2	0.0396 (17)	0.0422 (18)	0.0341 (16)	−0.0015 (14)	0.0112 (13)	−0.0054 (14)
N3	0.0515 (19)	0.0405 (19)	0.0335 (17)	0.0040 (16)	0.0088 (14)	0.0000 (14)
N4	0.0410 (17)	0.0374 (18)	0.0352 (16)	0.0000 (14)	0.0129 (13)	−0.0012 (14)
C1	0.056 (3)	0.054 (3)	0.047 (2)	−0.007 (2)	−0.002 (2)	−0.011 (2)
C2	0.043 (3)	0.071 (3)	0.068 (3)	−0.012 (2)	−0.007 (2)	−0.006 (3)
C3	0.037 (2)	0.067 (3)	0.069 (3)	0.001 (2)	0.011 (2)	0.014 (3)
C4	0.043 (2)	0.058 (3)	0.047 (2)	0.003 (2)	0.0191 (19)	0.003 (2)
C5	0.041 (2)	0.038 (2)	0.0325 (19)	−0.0045 (16)	0.0108 (16)	0.0054 (16)
C6	0.043 (2)	0.035 (2)	0.036 (2)	−0.0044 (17)	0.0074 (16)	0.0038 (16)
C7	0.040 (2)	0.035 (2)	0.0314 (18)	−0.0031 (16)	0.0113 (15)	0.0004 (15)
C8	0.058 (3)	0.045 (2)	0.042 (2)	0.010 (2)	−0.0002 (19)	−0.0038 (19)
C9	0.0336 (19)	0.045 (2)	0.041 (2)	−0.0077 (17)	0.0084 (16)	−0.0029 (18)
C10	0.069 (3)	0.045 (3)	0.047 (2)	0.009 (2)	0.016 (2)	0.004 (2)
C11	0.067 (3)	0.053 (3)	0.059 (3)	0.020 (2)	0.012 (2)	−0.004 (2)
C12	0.051 (3)	0.075 (3)	0.049 (3)	0.013 (2)	0.016 (2)	−0.013 (2)
C13	0.038 (2)	0.057 (3)	0.039 (2)	0.0047 (19)	0.0106 (16)	−0.0069 (19)
C14	0.047 (2)	0.079 (4)	0.045 (2)	0.003 (2)	0.0223 (19)	−0.001 (2)
C15	0.046 (2)	0.071 (3)	0.042 (2)	−0.003 (2)	0.0172 (19)	0.007 (2)
C16	0.0342 (19)	0.051 (2)	0.0332 (19)	−0.0069 (17)	0.0040 (15)	0.0030 (17)
C17	0.052 (2)	0.051 (3)	0.040 (2)	−0.007 (2)	0.0079 (18)	0.0110 (19)
C18	0.061 (3)	0.038 (2)	0.047 (2)	0.003 (2)	0.007 (2)	0.0075 (19)
C19	0.053 (2)	0.041 (2)	0.043 (2)	0.0037 (19)	0.0153 (19)	0.0003 (18)
C20	0.0297 (18)	0.043 (2)	0.0309 (18)	−0.0052 (16)	0.0031 (14)	−0.0026 (16)
C21	0.0360 (19)	0.044 (2)	0.0305 (18)	−0.0016 (16)	0.0050 (15)	−0.0051 (16)

### Geometric parameters (Å, °)

**Table:**

Cd1—O2^i^	2.189 (3)	C4—C5	1.391 (5)
Cd1—N2^i^	2.211 (3)	C4—H4A	0.930
Cd1—N1	2.236 (3)	C5—C6	1.397 (5)
Cd1—N4	2.327 (3)	C8—C9	1.530 (5)
Cd1—N3	2.405 (3)	C8—H8A	0.970
S1—C7	1.754 (4)	C8—H8B	0.970
S1—C8	1.811 (4)	C10—C11	1.384 (6)
O1—C9	1.225 (5)	C10—H10A	0.930
O2—C9	1.260 (5)	C11—C12	1.360 (7)
O2—Cd1^ii^	2.189 (3)	C11—H11A	0.930
N1—C7	1.344 (5)	C12—C13	1.400 (6)
N1—C6	1.387 (5)	C12—H12A	0.930
N2—C7	1.334 (5)	C13—C21	1.411 (5)
N2—C5	1.382 (5)	C13—C14	1.427 (6)
N2—Cd1^ii^	2.211 (3)	C14—C15	1.333 (6)
N3—C10	1.327 (5)	C14—H14A	0.930
N3—C21	1.347 (5)	C15—C16	1.427 (6)
N4—C19	1.323 (5)	C15—H15A	0.930
N4—C20	1.355 (5)	C16—C17	1.396 (6)
C1—C2	1.365 (6)	C16—C20	1.396 (5)
C1—C6	1.387 (5)	C17—C18	1.361 (6)
C1—H1A	0.930	C17—H17A	0.930
C2—C3	1.394 (7)	C18—C19	1.392 (6)
C2—H2A	0.930	C18—H18A	0.930
C3—C4	1.363 (6)	C19—H19A	0.930
C3—H3A	0.930	C20—C21	1.443 (5)
			
O2^i^—Cd1—N2^i^	104.44 (11)	C9—C8—S1	120.2 (3)
O2^i^—Cd1—N1	102.73 (12)	C9—C8—H8A	107.3
N2^i^—Cd1—N1	99.99 (11)	S1—C8—H8A	107.3
O2^i^—Cd1—N4	100.86 (11)	C9—C8—H8B	107.3
N2^i^—Cd1—N4	147.43 (11)	S1—C8—H8B	107.3
N1—Cd1—N4	94.03 (11)	H8A—C8—H8B	106.9
O2^i^—Cd1—N3	93.88 (12)	O1—C9—O2	124.8 (4)
N2^i^—Cd1—N3	87.71 (11)	O1—C9—C8	114.9 (4)
N1—Cd1—N3	159.18 (12)	O2—C9—C8	120.3 (4)
N4—Cd1—N3	70.30 (11)	N3—C10—C11	123.5 (4)
C7—S1—C8	102.5 (2)	N3—C10—H10A	118.2
C9—O2—Cd1^ii^	131.8 (3)	C11—C10—H10A	118.2
C7—N1—C6	103.6 (3)	C12—C11—C10	118.8 (4)
C7—N1—Cd1	123.9 (2)	C12—C11—H11A	120.6
C6—N1—Cd1	130.9 (2)	C10—C11—H11A	120.6
C7—N2—C5	104.4 (3)	C11—C12—C13	120.1 (4)
C7—N2—Cd1^ii^	119.8 (2)	C11—C12—H12A	120.0
C5—N2—Cd1^ii^	135.0 (3)	C13—C12—H12A	120.0
C10—N3—C21	118.2 (3)	C12—C13—C21	117.2 (4)
C10—N3—Cd1	126.8 (3)	C12—C13—C14	123.7 (4)
C21—N3—Cd1	114.9 (2)	C21—C13—C14	119.1 (4)
C19—N4—C20	117.9 (3)	C15—C14—C13	121.6 (4)
C19—N4—Cd1	124.2 (3)	C15—C14—H14A	119.2
C20—N4—Cd1	117.8 (2)	C13—C14—H14A	119.2
C2—C1—C6	117.7 (4)	C14—C15—C16	121.1 (4)
C2—C1—H1A	121.2	C14—C15—H15A	119.5
C6—C1—H1A	121.2	C16—C15—H15A	119.5
C1—C2—C3	121.8 (4)	C17—C16—C20	117.8 (4)
C1—C2—H2A	119.1	C17—C16—C15	122.6 (4)
C3—C2—H2A	119.1	C20—C16—C15	119.5 (4)
C4—C3—C2	121.3 (4)	C18—C17—C16	119.7 (4)
C4—C3—H3A	119.4	C18—C17—H17A	120.1
C2—C3—H3A	119.4	C16—C17—H17A	120.1
C3—C4—C5	117.5 (4)	C17—C18—C19	118.7 (4)
C3—C4—H4A	121.2	C17—C18—H18A	120.7
C5—C4—H4A	121.2	C19—C18—H18A	120.7
N2—C5—C4	130.7 (4)	N4—C19—C18	123.4 (4)
N2—C5—C6	108.1 (3)	N4—C19—H19A	118.3
C4—C5—C6	121.2 (4)	C18—C19—H19A	118.3
N1—C6—C1	131.0 (4)	N4—C20—C16	122.4 (4)
N1—C6—C5	108.5 (3)	N4—C20—C21	118.0 (3)
C1—C6—C5	120.5 (4)	C16—C20—C21	119.7 (3)
N2—C7—N1	115.5 (3)	N3—C21—C13	122.3 (4)
N2—C7—S1	122.9 (3)	N3—C21—C20	118.7 (3)
N1—C7—S1	121.5 (3)	C13—C21—C20	119.0 (4)

Symmetry codes: (i) −*x*, *y*+1/2, −*z*−1/2; (ii) −*x*, *y*−1/2, −*z*−1/2.
